# Evaluation of Apical Closure in Panoramic Radiographs Using Vision Transformer Architectures ViT-Based Apical Closure Classification

**DOI:** 10.3390/diagnostics15182350

**Published:** 2025-09-16

**Authors:** Sümeyye Coşgun Baybars, Merve Daldal, Merve Parlak Baydoğan, Seda Arslan Tuncer

**Affiliations:** 1 Department of Oral and Maxillofacial Radiology, Faculty of Dentistry, Fırat University, Elazığ 23000, Turkey; 2 Department of Computer Technologies, Fırat University, Elazığ 23000, Turkey; 3 Department of Software Engineering, Faculty of Engineering, Fırat University, Elazığ 23000, Turkey

**Keywords:** open apex, deep learning, panoramic radiograph, vision transformer

## Abstract

**Objective**: To evaluate the performance of vision transformer (ViT)-based deep learning models in the classification of open apex on panoramic radiographs (orthopantomograms (OPGs)) and compare their diagnostic accuracy with conventional convolutional neural network (CNN) architectures. **Materials and Methods**: OPGs were retrospectively collected and labeled by two observers based on apex closure status. Two ViT models (Base Patch16 and Patch32) and three CNN models (ResNet50, VGG19, and EfficientNetB0) were evaluated using eight classifiers (support vector machine (SVM), random forest (RF), XGBoost, logistic regression (LR), K-nearest neighbors (KNN), naïve Bayes (NB), decision tree (DT), and multi-layer perceptron (MLP)). Performance metrics (accuracy, precision, recall, F1 score, and area under the curve (AUC)) were computed. **Results**: ViT Base Patch16 384 with MLP achieved the highest accuracy (0.8462 ± 0.0330) and AUC (0.914 ± 0.032). Although CNN models like EfficientNetB0 + MLP performed competitively (0.8334 ± 0.0479 accuracy), ViT models demonstrated more balanced and robust performance. **Conclusions**: ViT models outperformed CNNs in classifying open apex, suggesting their integration into dental radiologic decision support systems. Future studies should focus on multi-center and multimodal data to improve generalizability.

## 1. Introduction

Tooth development is one of the fundamental biological indicators for both dental health and age estimation. The root development of permanent teeth begins after crown formation and ends with apical closure. Root development and apical closure are, according to Cvek, completed in five stages: (1) half root formation, (2) two-thirds root formation, (3) complete root formation with open apex, (4) complete root formation with partially closed apex, and (5) complete root formation with closed [1]. Teeth with open apices present challenges in endodontic, orthodontic, and surgical treatment planning and provide essential information on pulp vitality and root development [2,3]. Systemic diseases, trauma, or pulpal infections during root formation can negatively affect apical closure and complicate treatment planning. The early detection of resorptive changes that may compromise root integrity is also important, as such damage can worsen prognosis and make treatment more complex [4,5].

Orthopantomographs (OPGs) are among the most frequently used imaging techniques for evaluating open apices, as they provide a broad anatomical overview with low radiation exposure and cost effectiveness. Several studies have similarly employed panoramic radiographs as the primary modality for assessing root development and apical status, underlining their routine use in both clinical and research settings [6]. For instance, Patel et al. (2020) demonstrated that, while certain radiographic signs on OPG correlate with cone beam computed tomography (CBCT) findings in the evaluation of third molar–inferior alveolar nerve relationships, in the absence of such signs OPG alone was often sufficient [7]. Likewise, Kalabalık et al. (2024), in their investigation of root apical closure, have emphasized that, although CBCT provides detailed three-dimensional visualization, its considerably higher radiation dose compared with OPG limits its appropriateness for routine assessments, particularly in children [8]. In contrast, CBCT, although offering three-dimensional visualization, is associated with substantially higher radiation exposure, increased cost, longer acquisition and interpretation times, and limited accessibility. Moreover, its application is generally limited to cases with specific diagnostic indications and is not recommended for routine evaluation of root development in asymptomatic populations [8,9]. Considering these disadvantages, OPG was selected as the primary imaging modality in this study, reflecting both clinical practice standards and the feasibility of retrospective data analysis.

Despite these advantages, evaluating apical details on OPGs may be limited by image quality, projection errors, and anatomical superimpositions. The detection of open apices can be particularly challenging, as it often depends on the observer’s clinical experience and attentiveness, and factors such as fatigue and technical variations during image acquisition may further complicate assessment. Variability between observers and time-consuming manual analysis can make diagnosis more difficult [10,11]. There is an increasing demand for objective, rapid, and reliable methods for classifying root development and apical closure. The accurate and consistent identification of open apices is especially important, as it can directly influence treatment planning and care protocols; therefore, AI-based approaches hold promise for improving clinical decision-making by enabling faster and more reliable detection of open apex cases [12].

Among deep learning models, CNNs are widely used in dental image classification and segmentation but can be limited in capturing long-range dependencies [13,14]. Recently, vision transformer (ViT)-based models have emerged as an alternative for image classification and analysis [15,16]. Unlike traditional CNNs, transformer architectures use a self-attention mechanism to capture global relationships across the entire image. This enables ViT models to learn long-range dependencies and complex structures more effectively, offering advantages for dental images with significant morphological variation [16,17]. ViTs divide input images into fixed-size patches and optimize the representation of each patch using attention mechanisms to form a holistic image representation [15]. In dental radiology, the applications of this architecture remain limited and, despite growing evidence, are still in their early stages [17]. Recent studies have emphasized the value of lightweight attention mechanisms in improving boundary fidelity and capturing thin anatomical structures. For example, Zhang et al. have demonstrated that ViTs outperform CNNs in furcation involvement classification on panoramic radiographs by better modeling subtle structural variations [18]. Similarly, Kahm et al. showed that analyzing entire panoramic images for age estimation benefits from multi-scale contextual features beyond isolated teeth [19]. In medical imaging, lightweight attention-based architectures such as Attention GhostUNet++ have been shown to refine fine boundaries while maintaining computational efficiency, and super-resolution approaches in endoscopic imaging further highlight the role of transformer-based attention in preserving detail and enhancing multi-scale context [20,21].

The timely and objective assessment of open apices is important for accurate treatment planning and prognosis. AI-based automated methods have the potential to provide faster and more consistent results when compared with traditional evaluations, thereby supporting clinical decision-making. Therefore, the present study aims to determine the performance of ViT Base Patch32 and ViT Base Patch16 models in classifying open apices in dental OPGs and to conduct a comparative analysis with CNN architectures, evaluating the potential contributions of next-generation models to clinical applications.

## 2. Materials and Methods

### 2.1. Dataset

Panoramic radiographs used in this study were retrospectively reviewed based solely on image data, with no patient demographic information (e.g., age or gender) considered. Images were obtained from patients who underwent panoramic imaging for various clinical indications at the Department of Oral and Maxillofacial Radiology, Faculty of Dentistry, Fırat University, and had provided informed consent for scientific use. To ensure consistency and to enable the assessment of open apex status in both jaws, only single-rooted teeth from the maxilla and mandible were included in the study.

**Figure 1:**
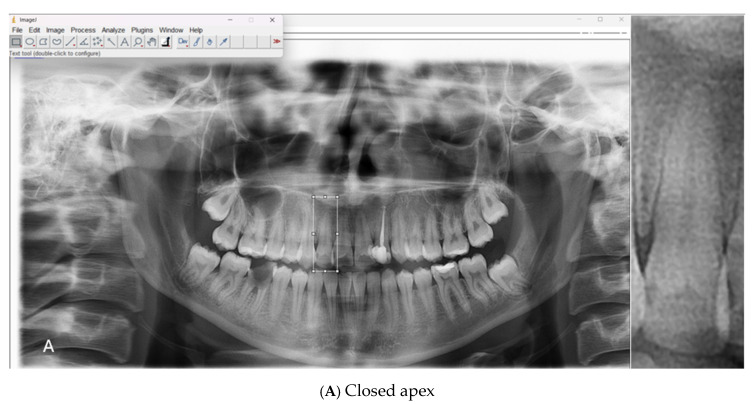

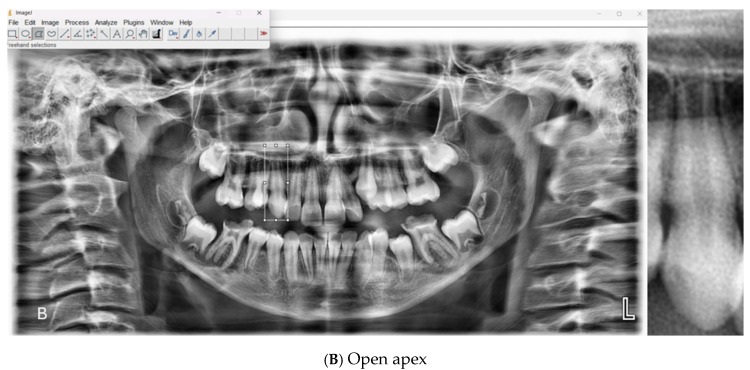
Representative panoramic radiographs and cropped regions of interest (ROIs) showing (**A**) closed apex and (**B**) open apex teeth.

All scans were acquired using a Carestream Dental CS 8100 unit (LLC, 2019) under standard exposure protocols (68 kV, 8 mA, 66 mGy·cm^2^, 10.8 s). The original images, stored in JPEG format, were processed using ImageJ software (version 1.54g, National Institutes of Health, Bethesda, MD, USA) The apical regions of the relevant teeth were manually isolated, and the regions of interest (ROIs) were saved in PNG format. A total of 1008 ROI images were included in the dataset, consisting of 500 open apex (class 1) and 508 closed apex (class 2) images (Table 1). Representative examples of panoramic radiographs and cropped ROI images used in this study are shown in Figure 1.

To assess inter-observer agreement, 100 panoramic radiographs were independently labeled by two observers: one oral and maxillofacial radiology specialist with ≥5 years of experience and one research assistant with 3 years of experience. Cohen’s Kappa analysis was performed using SPSS software (version 22.0, IBM Corp., Armonk, NY, USA)yielding a kappa value of 0.80, which indicates a substantial level of agreement between observers. In the main dataset, root apex status was jointly evaluated by both observers through simultaneous and consensus-based decision-making. Images with inadequate quality, indistinct apical regions due to superimposition, or unclear anatomical structures—as well as those on which consensus could not be reached—were excluded from the study. As a result of these exclusions, a total of 1008 ROI images were included in the final dataset.

All ROI images were resized to 224 × 224 pixels, and pixel values were normalized to a range of 0–1. The images were retained in RGB format, and grayscale conversion was intentionally avoided to preserve spatial detail and maintain compatibility with both vision transformer (ViT) and CNN-basedmodels. To improve the model’s generalization capability and reduce the risk of overfitting, data augmentation techniques such as horizontal flipping and minor angular rotations were applied during the training phase. All deep learning models, including ViT and CNN architectures, were trained and validated on the same standardized dataset to ensure a fair and consistent comparison of performance.

### 2.2. Proposed Method—ViT Architecture and Utilized Models

In the field of image classification, the ViT is recognized in the literature as a powerful deep learning architecture based on attention mechanisms, with a strong capacity for effective feature extraction. Within the scope of this study, feature extraction and classification processes were carried out using ViT Base Patch32 and ViT Base Patch16 models to ensure accurate classification of open apex in dental radiographs. The ViT Base Patch32 model divides the input image into 32 × 32-sized patches, converting each patch into a fixed-size vector; the ViT Base Patch16 model performs the same operation with 16 × 16-sized patches. These vectors are passed to the model through linear embedding layers, and the relationships between each patch and all other patches are learned using a multi-head self-attention mechanism. The parameters and characteristics of the models and algorithms used in this study are presented in Table 2.

As an output of the model, a 1000-dimensional feature vector is obtained. In the ViT Base Patch32 model, fewer patches are generated, which leads to lower computational cost and more efficient memory usage. This makes it advantageous for achieving fast and sufficient performance in less complex visual tasks or in environments with limited hardware resources. However, the reduced number of patches may result in a lower level of detail, making the Patch32 model more effective when recognizing general visual patterns. In contrast, the Patch16 model is preferred for classification tasks that require higher detail and resolution.

**Figure 2:**
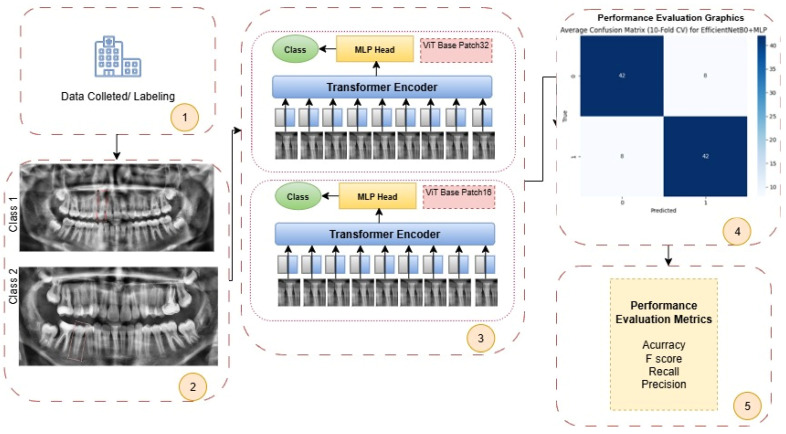
Workflow diagram illustrating the step-by-step process of data collection, labeling, ViT model application (Patch32 and Patch16), performance evaluation, and metric calculation for open apex classification from panoramic radiographs.

In this study, the performance of the open apex classification task performed using ViT Base Patch32 and ViT Base Patch16 models was analyzed. The classification performance obtained was compared with conventional CNN-based models, specifically ResNet50, EfficientNetB0, and VGG19, applied on the same dataset. As a result of this comparison, the superiority of the ViT architecture over traditional CNN-based approaches in the classification of open apex was demonstrated using objective performance metrics. All operational steps of the proposed ViT-based system are schematically presented in Figure 2.

#### Step-by-Step Processing of Each Patient’s Root Apex Status

Step 1: In the initial phase of the study, OPGs were collected from various healthcare institutions. The acquired images were evaluated by expert clinicians and labeled based on open apex status.

Step 2: The labeled radiographic images were categorized by the experts into two classes according to the presence or absence of an open apex: Class 1 (closed apex) and Class 2 (open apex). This classification ensured correct guidance of input data during the training phase of the deep learning models.

Step 3: The images were input into ViT-based architectures (ViT Base Patch32 and ViT Base Patch16). In these architectures, images were divided into patches of size 32 × 32 and 16 × 16, respectively. These patches were processed through transformer encoder blocks to extract 1000-dimensional feature representations. Following feature extraction, classification was performed using a multi-layer perceptron head.

Step 4: Based on the classification results, various performance plots and curves (e.g., ROC curve, confusion matrix) were generated for visual evaluation of model performance. These visualizations provide an intuitive understanding of the model’s overall behavior and classification accuracy.

Step 5: The model’s performance was quantitatively evaluated using key metrics such as accuracy, F1 score, recall, and precision, assessing the ability to distinguish correctly between both positive and negative classes.

OPGs, which are commonly used in contemporary clinical practice, are crucial imaging tools for the general evaluation of dental structures and root morphology. However, accurately determining open apex based on these images can be significantly affected by technical factors such as image quality and projection angle, as well as observer-dependent interpretation variability. In recent years, the ViT architecture has gained prominence in the field of computer vision due to its ability to process each patch of an input image individually. This capability allows for the extraction of high-dimensional and semantically rich features from OPGs, enabling more precise analysis of structural variations in root morphology that are difficult to detect visually.

### 2.3. Comparative Models and Evaluation

In order to comprehensively evaluate the performance of the ViT models, traditional CNN-based approaches were also applied to the same dataset, and comparative analyses were conducted. The performance of the ViT models was compared with classical CNN-based architectures, specifically ResNet50, EfficientNetB0 and VGG19.

To assess the performance of these models, a confusion matrix was used. From the confusion matrix, performance metrics such as accuracy, recall, precision, specificity, and F1 score were calculated. The formulas used for computing these metrics are presented below (Equations (1)–(4)):

Accuracy = (TP + TN)/(TP + TN + FP + FN)(1)

Recall = TP/(TP + FN)(2)

Precision = TP/(TP + FP)(3)

F1 score = 2TP/(2TP + FN + FP)(4)

In the formulas above, TP = true positive, TN = true negative, FP = false positive, and FN = false negative.

### 2.4. Data Availability and Ethical Considerations

This study was designed as a retrospective analysis. Panoramic radiographs used in this research were obtained from the digital archive of Fırat University Faculty of Dentistry, covering the period between 2023 and 2024. As the data were retrospectively collected and anonymized prior to analysis, individual patient consent for publication was not required. However, all patients had provided written informed consent at the time of admission for the anonymous use of their data for scientific purposes.

Due to institutional data protection regulations, the full dataset (including raw radiographic images) is not publicly available. However, de-identified image samples or further technical information can be made available by the corresponding author upon reasonable request for academic and non-commercial purposes.

This study received approval from the Non-Interventional Research Ethics Committee of Fırat University (Decision No: 2024/15-16, dated 19 December 2024).

### 2.5. Use of Generative AI

Generative artificial intelligence tools (OpenAI’s ChatGPT-4) were used exclusively for language editing and paraphrasing of English-language content to improve grammar, clarity, and academic tone. No AI-based tools were utilized for data generation, image creation, statistical analysis, or study design.

## 3. Results

In order to evaluate the performance of the artificial intelligence (AI)-based classification system developed in this study for apex closure status classification (open vs. closed), both conventional convolutional neural network (CNN) architectures and ViT-based models were tested on the same dataset. ResNet50, EfficientNetB0, and VGG19 were selected as the CNN architectures, while ViT Base Patch32 and ViT Base Patch16 were employed as the ViT-based models. Model performance was assessed using standard classification metrics—accuracy, recall, specificity, precision, and F1 score—derived from confusion matrices. Ten-fold cross-validation was employed to ensure robust evaluation.

In the first stage, apex closure status classification (open vs. closed) was performed using ResNet50, VGG19, and EfficientNetB0 architectures in combination with eight machine learning classifiers (SVM, RF, XGBoost, LR, KNN, NB, DT, and MLP). The ResNet50 model achieved its best performance with the MLP classifier (accuracy = 0.7986 ± 0.0337, F1 score = 0.7978 ± 0.0340), followed by LR (accuracy = 0.7570 ± 0.0447) and XGBoost (accuracy = 0.7451 ± 0.0406). In contrast, NB (accuracy = 0.6787 ± 0.0613) and DT (accuracy = 0.6072 ± 0.0477) yielded lower results. The VGG19 architecture produced more balanced outcomes; in particular, MLP (accuracy = 0.8036 ± 0.0211, F1 score = 0.8033 ± 0.0215) and SVM (accuracy = 0.8016 ± 0.0485, F1 score = 0.8011 ± 0.0492) stood out, while XGBoost (accuracy = 0.7659 ± 0.0209) and RF (accuracy = 0.7619 ± 0.0346) also provided satisfactory results. Overall, EfficientNetB0 delivered the highest performance, with SVM (accuracy = 0.8334 ± 0.0379, F1 score = 0.8330 ± 0.0383) and MLP (accuracy = 0.8334 ± 0.0479, F1 score = 0.8332 ± 0.0479) combinations achieving the best outcomes. These findings suggest that, due to its richer parameterization, EfficientNetB0 outperformed the other CNN architectures in classifying apex closure status. Although ResNet50 also produced competitive results, particularly with MLP and LR classifiers, its overall performance was lower than that of EfficientNetB0 (Table 3).

CNN-based models are limited in contextual awareness because they primarily rely on local feature extraction. To overcome these constraints, the ViT architecture, with its ability to model global context in visual data, was evaluated as an alternative approach. Both ViT Base Patch32 and Patch16 models were tested at two input resolutions (224 and 384) and consistently demonstrated higher performance compared with CNN-based models. For the ViT-base-patch16-224 model, the MLP classifier achieved the highest accuracy (0.8085 ± 0.0320), while SVM (0.8016 ± 0.0385) and LR (0.7956 ± 0.0350) also performed strongly. With the ViT Base Patch32-224 model, the best results were again obtained with MLP (0.8244 ± 0.0387), followed by SVM (0.8115 ± 0.0464) and LR (0.7817 ± 0.0428). At the higher resolution, the ViT Base Patch 16-384 model reached the highest overall accuracy (0.8462 ± 0.0330) with MLP, with SVM (0.8145 ± 0.0433) and LR (0.8065 ± 0.0440) also producing competitive outcomes. Similarly, the ViT Base Patch32-384 model performed well, with MLP (0.8402 ± 0.0289), SVM (0.8243 ± 0.0372), and LR (0.8223 ± 0.0364) standing out. Overall, ViT models showed superior performance compared with CNN-based architectures, particularly when smaller patch sizes (Patch16) and higher resolutions (384) were used. These results indicate that the attention-based structure of ViT provides more effective classification capacity in clinical decision-making tasks such as apex closure evaluation (Table 4).

**Figure 3:**
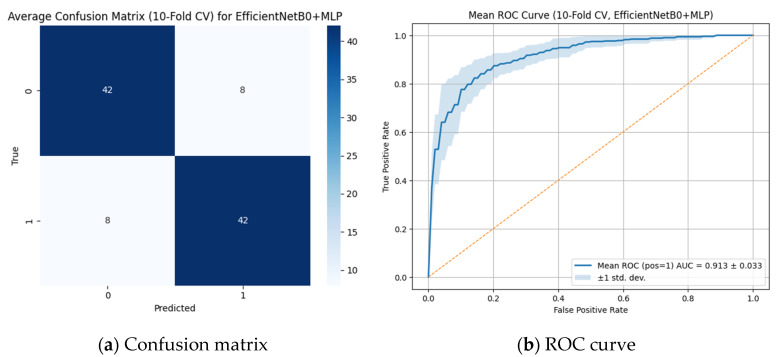
Best-performing traditional CNN model (EfficientNetB0 + MLP) in classifying open apex status. (**a**) Confusion matrix showing 42 correct predictions for each class, indicating balanced classification performance across open and closed apex categories. (**b**) ROC curve with an AUC of 0.913, reflecting a strong discriminative ability of the EfficientNetB0 + MLP model with consistent performance across cross-validation folds.

The classification performance of the model for detecting open apex was evaluated using the CNN-based best-performing EfficientNetB0 + MLP model based on the ROC curve (Figure 3a) and the confusion matrix (Figure 3b).

As shown in Figure 3, the average confusion matrix obtained through 10-fold cross-validation demonstrated that the EfficientNetB0 + MLP model correctly classified both positive and negative classes with an overall accuracy of 84% (TP = 42, TN = 42, FP = 8, FN = 8). The mean area under the ROC curve (AUC) was 0.913 ± 0.033, indicating high discriminative ability in distinguishing between classes. The low standard deviation of the AUC further suggests consistent performance across different data subsets. These results highlight the effectiveness and reliability of the EfficientNetB0 + MLP-based approach for sensitive medical imaging tasks such as apex closure evaluation.

**Figure 4:**
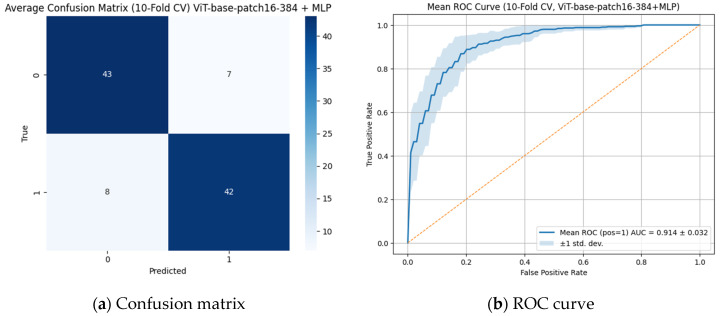
Best performing vision transformer model (ViT Base Patch 16-384 + MLP) in classifying open apex status. (**a**) Confusion matrix showing 42 correct predictions for open apex and 43 for closed apex, demonstrating balanced and accurate classification performance. (**b**) ROC curve with an AUC of 0.914, indicating a high discriminative ability of the model with consistent performance across cross-validation folds.

As analyzed in Figure 4, when assessed with 10-fold cross-validation, the model demonstrated high classification performance. The average confusion matrix showed that the model classified the positive class with 84% accuracy and the negative class with 86% accuracy (TP = 42, TN = 43, FP = 7, FN = 8). The overall accuracy of the model was calculated as 85%, while the precision and recall values were 0.857 and 0.84, respectively. In addition, the area under the ROC curve (AUC) was determined as 0.914 ± 0.032, indicating that the model possesses a very high discriminative ability in distinguishing between classes. The low standard deviation further demonstrates that the model produced consistent results across different data subsets; therefore, this ViT-based approach emerges as an effective and reliable method for detecting critical medical structures such as open apex. In this context, although no statistically significant difference was observed, the ViT Base Patch 16-384 + MLP model can be considered to have outperformed CNN-based models, likely due to its attention-based global feature extraction capabilities.

**Figure 5:**
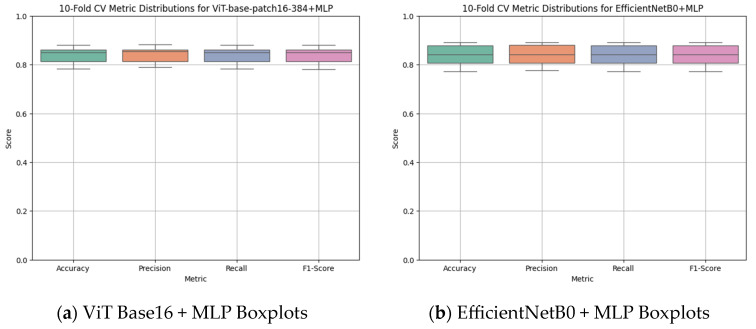
Ten-fold cross-validation metric distributions for (**a**) ViT Base Patch 16-384 + MLP and (**b**) EfficientNetB0 + MLP models in the classification of open apex status. The boxplots display the distributions of accuracy, precision, recall, and F1 score across folds, demonstrating that the ViT-based model achieved slightly higher and more consistent performance across all metrics.

According to the 10-fold cross-validation results presented in Figure 5, the ViT Base Patch 16-384 + MLP model (Figure 5a) demonstrated higher and more consistent performance across all fundamental classification metrics compared with the EfficientNetB0 + MLP model (Figure 5b). For the ViT-based model, the averages of accuracy, precision, recall, and F1 score clustered within the 0.85–0.88 range, and the compact box plots indicated low variance, confirming stable performance across folds. In contrast, although the EfficientNetB0 + MLP model also produced relatively balanced outcomes, a wider spread was observed in some metrics, particularly recall. Overall, these findings suggest that the ViT-based approach not only achieves higher accuracy in apex closure status classification but also provides more reliable and stable outputs throughout cross-validation.

**Figure 6:**
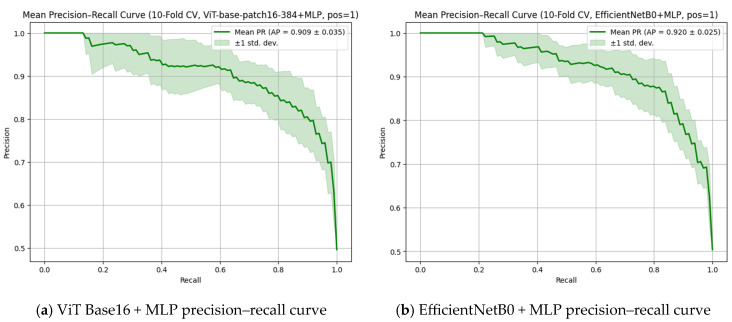
Precision–recall (PR) curves for (**a**) ViT Base Patch 16-384 + MLP and (**b**) EfficientNetB0 + MLP models in open apex classification, illustrating high performance from both models with slightly higher average precision in the EfficientNetB0 model and more consistent behavior in the ViT-based model. Statistical analysis.

Figure 6 presents the precision–recall (PR) curves for the ViT Base Patch 16-384 + MLP (Figure 6a) and EfficientNetB0 + MLP (Figure 6b) models, both of which demonstrated high discriminative performance in apex closure status classification. The EfficientNetB0 + MLP model achieved a slightly higher average precision (AP) score of 0.920 ± 0.025, whereas the ViT-based model reached a comparable AP score of 0.909 ± 0.035, reflecting strong overall accuracy despite a slightly wider variance across folds. Although the numerical AP score favored EfficientNetB0, when considering the overall classification metrics, consistency, and the visual patterns of the PR curves, the ViT-based model can be regarded as offering superior overall performance.

**Figure 7:**
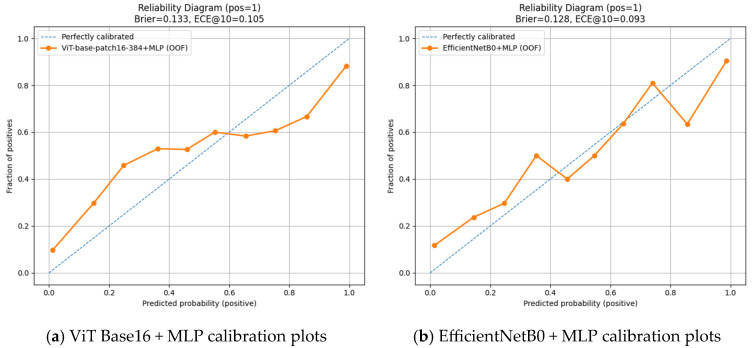
Calibration curves for (**a**) ViT Base Patch 16-384 + MLP and (**b**) EfficientNetB0 + MLP models in open apex classification, showing better calibrated probability estimates in the EfficientNetB0 model based on lower Brier and ECE scores.

Figure 7 presents the calibration plots (CP) for the ViT Base Patch 16-384 + MLP (Figure 7a) and EfficientNetB0 + MLP (Figure 7b) models. The EfficientNetB0 + MLP model provided better calibrated probability estimates, as reflected by a lower Brier score (0.128) and ECE@10 value (0.093). However, this advantage in calibration does not translate into superior overall classification performance. Considering all other evaluation metrics—accuracy, precision, recall, F1 score, AUC, AP, and variance analysis—the ViT Base Patch 16-384 + MLP model achieved higher accuracy, stronger discriminative ability, and more consistent results. Thus, while the ViT-based model outperforms in overall classification success, the EfficientNetB0-based model demonstrates a more favorable profile specifically in probability calibration.

## 4. Discussion

Open apex plays a critical role in endodontic, orthodontic, and surgical treatment planning and is widely used to assess whether root development has been completed [22]. However, the evaluation of open apex on OPGs may be subject to interpretive errors due to technical limitations such as superimposition and image quality [23]. Numerous studies in the literature have demonstrated that the stage of root development is a key factor not only in determining the appropriate treatment protocol but also in ensuring long-term functional success, underlining the importance of precise and timely radiographic assessment, especially when evaluating open and closed apices. Shobhana et al. elaborated on the clinical challenges encountered in apexification procedures for teeth with incomplete root development, highlighting that thin root walls and open apices limit the effectiveness of canal disinfection, shaping, and obturation procedures—rendering the treatment technically more complex [22]. They further emphasized that the biocompatibility of the materials used and their ability to form an apical barrier are critical factors directly affecting treatment success. Similarly, in a study conducted by Kumar et al., it was reported that periapical healing was achieved in all cases following single-visit apexification using different techniques. However, the authors stressed that strict adherence to treatment protocols plays a significant role in reducing the risk of complications [24]. These findings demonstrate that, for teeth with immature roots and open apical foramina, accurate and timely radiographic identification is essential—not only for successful treatment planning but also for biological healing and long-term prognosis. Therefore, inaccurate or delayed classification of open apices may increase the complexity of treatment protocols, elevate the technical challenges of clinical procedures, and adversely affect long-term outcomes; highlighting the critical importance of precise and timely radiographic assessment [22,24,25].

CNN-based models, which are among the traditional deep learning architectures, have long been successfully applied in the analysis of dental radiographs and are considered a foundational reference in the field [26]. However, ViT-based architectures, which have emerged in recent years, have stood out as strong alternatives—particularly due to their ability to model contextual relationships and achieve high accuracy even with limited datasets. For this reason, in order to better assess the potential of ViT models in clinical radiological diagnostics, it is beneficial to examine findings from previous studies based on CNN-based approaches [27].

In the study by Mun et al., the researchers evaluated the applicability of deep learning models for determining extraction indications in cracked teeth using OPGs. They achieved sensitivity rates above 90% and F1 scores in the range of 76–79% with InceptionV3, ResNet50, and EfficientNetB0 architectures. These results suggest that deep learning models can offer clinically meaningful diagnostic accuracy and serve as effective decision support tools in the diagnosis of cracked teeth [28]. Similarly, in a study conducted by Pirayesh et al., deep learning models were used for the automatic diagnosis of canine-induced root resorption and effective outcomes were achieved even with a limited number of cone beam computed tomography (CBCT) datasets. Among the five tested strategies, Model C (classification with 3D ResNet) and Model E (classification using only the encoder part of a pre-trained model) demonstrated the best performance, with 82% accuracy and an F1 score of 0.62. These findings indicate that AI-based approaches using volumetric data may offer higher diagnostic success in detecting root resorption compared with conventional methods [29]. Consistently, in our study, CNN-based models such as ResNet50, EfficientNetB0 and VGG19 were evaluated with various classifiers, and F1 scores ranged from 61% to 83%. The highest F1 score (≈83%) was obtained with the EfficientNetB0–MLP combination, while ResNet50 and VGG19 also produced competitive results (≈80%). These findings align with the observations of Pirayesh et al. and Mun et al., supporting the results reported in the literature.

The findings obtained using CNN-based architectures highlight the applicability of deep learning-based classification approaches in dental radiology. However, the ViT architecture, which has gained increasing attention in recent years for image classification tasks, is emerging as a powerful alternative—particularly in the analysis of localized anatomical structures [30]. In this context, the following section presents a comparative evaluation of ViT-based models and conventional CNN architectures to explore the impact of different deep learning approaches on diagnostic accuracy.

Shisu et al. compared their proposed EATFormer model for chest radiograph analysis with various CNN-based (DenseNet, ResNetV2, EfficientNet, Xception, Inception) and transformer-based (MedViT, DeiT-Ti, Swin, LocalViT) architectures, reporting the highest performance with an accuracy of 95.3%. Ahmed et al. achieved an F1 score of 97% by combining the ViT architecture with GRU for brain tumor detection on MRI images, demonstrating the superiority of this hybrid model over CNN-based models such as DenseNet121, ResNet18, and VGG19 [31,32]. In the present study, the classification performance of ViT-based models (Patch 16 and Patch 32) and CNN-based models was compared in the context of apex closure status classification. The results revealed that the ViT Base Patch 16-384 model achieved the highest accuracy at 0.8462 (≈85%), while CNN-based models yielded accuracy rates ranging between 0.6072 (≈61%) and 0.8334 (≈83%). These findings indicate that the transformer architecture, owing to its capacity to model long-range dependencies, is more effective at capturing morphological variations in dental radiographs. Moreover, in our study, ViT models demonstrated more consistent results than CNN-based models in terms of both classification accuracy and class balance.

Although the ViT architecture has so far been applied in a limited number of studies within the field of dentistry, promising results have been reported in various diagnostic tasks. Yang et al. developed the ImplantFormer model for implant position detection and compared it with CNN-based methods such as Faster R-CNN, Cascade R-CNN, and CenterNet. They reported that the ViT Base ResNet-50 model achieved the highest performance with an AP75 value of 13.7% [33]. Similarly, in the present study, the ViT Base Patch 16-384 model achieved the highest classification accuracy—up to 0.8462 (≈85%) in the assessment of apex closure status on OPGs, outperforming conventional CNN architectures. These findings support the growing body of evidence that ViT-based models offer superior diagnostic performance across a range of dental imaging tasks.

Zhang et al. used the MC-ViT architecture for the detection of furcation involvement and achieved impressive results, including 92% accuracy, 98% precision, 92% recall, and a 95% F1 score—demonstrating that ViT models significantly outperformed traditional methods in OPG analysis. These results highlight the capacity of ViT models to capture subtle anatomical features in dental imaging [18]. Similarly, in our study, the ViT Base Patch 16-384 model achieved the highest classification accuracy of 0.8462 (≈85%) in the assessment of apex closure status. However, while Zhang et al. worked with more localized and high-resolution images, our study was conducted using OPGs, which are more prone to lower resolution and anatomical superimpositions. Despite these challenges, the comparable level of accuracy observed in our results underscores the robustness of the ViT architecture in complex imaging conditions.

Felsch et al. demonstrated the successful use of ViT models in the segmentation of dental caries and MIH lesions, reporting high levels of diagnostic accuracy [34]. Likewise, our study showed that the ViT Base Patch 16-384 model achieved a high accuracy of 0.8462 (≈85%) in classifying apex closure status on OPGs, further confirming the potential of this architecture in dental radiology. Nevertheless, the photographic images used in Felsch et al.’s study offered higher visual clarity and color contrast, whereas our work was limited to grayscale OPGs with lower spatial resolution. The fact that comparable classification performance was still achieved suggests that the ViT architecture has strong adaptability across different imaging modalities.

Dujic et al. applied ViT, BEiT, and DeiT models to periapical radiographs for the detection of periodontal bone loss, reporting overall accuracy between 83.4% and 85.2%, and AUC values ranging from 0.899 to 0.918 [35]. These findings closely align with the results of the present study, where the ViT Base Patch 16-384 model achieved up to 0.8462 (≈85%) accuracy, supporting the reliability of transformer-based models in dental diagnostics. However, while Dujic et al. used periapical images—which are more suitable for localized assessments and offer higher resolution—our study was conducted on OPGs, which cover broader anatomical regions but are more susceptible to distortion and superimposition. The ability of the ViT model to perform reliably under these limitations further emphasizes its generalizability across different dental imaging types.

In conclusion, ViT-based models demonstrated high accuracy and generalizability in classifying root development on OPGs —despite the low resolution and complex structural features of such images—outperforming conventional CNN models. These findings support the growing application potential of the ViT architecture in dental imaging and indicate that it can contribute significantly to clinical decision support systems.

### Strengths, Limitations and Future Directions

The most important strength of this study is the application of the ViT, a contemporary deep learning architecture, for the classification of apical closure in panoramic radiographs. The use of this architecture, which is relatively new in the dental literature, enhances the originality of the study. Furthermore, the direct comparison of ViT models with CNN-based architectures highlights the performance differences between these two approaches, providing a meaningful contribution to the literature. Another strength of the study is its focus on the evaluation of open apices using artificial intelligence, which has the potential to support clinicians in treatment planning and facilitate clinical decision-making. Finally, the use of panoramic radiographs as the primary imaging modality, due to their widespread availability in clinical practice and ability to provide a broad anatomical overview in a single exposure, increases the clinical relevance of the study and contributes to standardization in evaluations.

Nevertheless, this study has several limitations. It was conducted at a single center using only one panoramic device, which restricts the generalizability of the findings. Furthermore, the study focused exclusively on evaluating the classification performance of deep learning models; clinical utility measures such as time saved per case compared with manual inspection or misclassification cost analysis were not included within the scope. Due to the limited sample size, performance differences between ViT models and CNN models could not be formally tested for statistical significance. Instead, effect size estimates, class-wise sensitivity/specificity values, and confidence intervals were reported.

Future research should be carried out on larger, multi-center datasets acquired from different imaging devices to enhance the external validity of the findings. In addition, the assessment of clinical utility measures, such as time saved per case and the clinical consequences of misclassifications, will be essential for demonstrating the real-world effectiveness of these models. Moreover, integrating ViT architectures with three-dimensional imaging modalities such as CBCT could provide valuable insights into their diagnostic performance across different imaging techniques and facilitate their integration into routine clinical practice.

## 5. Conclusions

This study demonstrated the effectiveness of ViT-based deep learning models for the automatic classification of open apex in dental OPGs. The ViT Base Patch 16 model achieved higher accuracy, precision, and recall compared with traditional CNN-based models such as ResNet50, EfficientNetB0 and VGG19. These results suggest that the ViT architecture represents a significant advancement in the integration of medical image analysis into clinical decision support systems. In the future, the expansion of the dataset and the application of multimodal learning strategies are expected to further enhance model performance.

## Figures and Tables

**Table 1:** Distribution of the dataset across classes.

Data Set	Count
**Open apex (Class 1)**	500
**Closed apex (Class 2)**	508
**Total**	1008

**Table 2:** Parameters and characteristics of the models and algorithms used in the study.

Algorithm/ Model	Metrics and Characteristics
ResNet50	Params: 25.56M, GFLOPs: 4.13, Latency(ms/img): 184.40, Peak VRAM (GB): N/A CPU
VGG19	Params: 143.67M, GFLOPs: 19.69, Latency(ms/img): 742.74, Peak VRAM (GB): N/A CPU
EfficientNetB0	Params: 5.29M, GFLOPs: 0.41, Latency(ms/img): 49.56, Peak VRAM (GB): N/A CPU
ViT-B/16(224)	Params: 86.57M, GFLOPs: 12.02, Latency(ms/img): 690.87, Peak VRAM (GB): N/A CPU
ViT-B/16(384)	Params: 86.86M, GFLOPs: 39.27, Latency(ms/img): 1886.25, Peak VRAM (GB): N/A CPU
ViT-B/32(224)	Params: 88.22M, GFLOPs: 3.00, Latency(ms/img): 205.12, Peak VRAM (GB): N/A CPU
ViT-B/32(384)	Params: 88.30M, GFLOPs: 8.96, Latency(ms/img): 488.66, Peak VRAM (GB): N/A CPU
KNN	n_neighbors = 5, weights = ‘uniform’, algorithm = ‘auto’, leaf_size = 30, p = 2, metric = ‘minkowski’, others = default
SVM	C = 1.0, kernel = ‘rbf’, degree = 3, gamma = ‘scale’, coef0 = 0.0, probability = False, others = default
NB	var_smoothing = 1 × 10^−9^, priors = None, others = default
DT	Criterion = ‘gini’, splitter = ‘best’, min_samples_split = 2, min_samples_leaf = 1, others = default
MLP	hidden_layer_sizes = (100), activation = ‘relu’, solver = ‘adam’, alpha = 0.0001, max_iter = 200, others = default
LR	Penalty = ‘l2’, C = 1.0, solver = ‘lbfgs’, max_iter = 100, multi_class = ‘auto’, others = default
RF	n_estimators = 100, criterion = ‘gini’, max_depth = None, min_samples_split = 2, others = default
XGBoost	n_estimators = 100, learning_rate = 0.1, max_depth = 3, subsample = 1, others = default

**Table 3:** Performance evaluation metrics obtained from CNN-based models.

Model	Classifier	Performance Evaluation Metrics			
		Precision	Recall	F1 Score	Accuracy
ResNet50	SVM	0.7434 ± 0.0485	0.7283 ± 0.0459	0.7246 ± 0.0470	0.7292 ± 0.0456
	RF	0.7431 ± 0.0538	0.7404 ± 0.0540	0.7393 ± 0.0547	0.7401 ± 0.0542
	XGBoost	0.7476 ± 0.0394	0.7451 ± 0.0404	0.7443 ± 0.0411	0.7451 ± 0.0406
	LR	0.7584 ± 0.0448	0.7569 ± 0.0449	0.7566 ± 0.0449	0.7570 ± 0.0447
	KNN	0.7434 ± 0.0485	0.7283 ± 0.0459	0.7246 ± 0.0470	0.7292 ± 0.0456
	NB	0.6809 ± 0.0618	0.6786 ± 0.0612	0.6776 ± 0.0616	0.6787 ± 0.0613
	DT	0.6089 ± 0.0492	0.6072 ± 0.0475	0.6060 ± 0.0473	0.6072 ± 0.0477
	MLP	0.8031 ± 0.0337	0.7985 ± 0.0339	0.7978 ± 0.0340	0.7986 ± 0.0337
VGG19	SVM	0.8036 ± 0.0463	0.8017 ± 0.0482	0.8011 ± 0.0492	0.8016 ± 0.0485
	RF	0.7649 ± 0.0345	0.7621 ± 0.0346	0.7613 ± 0.0347	0.7619 ± 0.0346
	XGBoost	0.7665 ± 0.0206	0.7659 ± 0.0208	0.7657 ± 0.0209	0.7659 ± 0.0209
	LR	0.7406 ± 0.0250	0.7380 ± 0.0267	0.7372 ± 0.0273	0.7381 ± 0.0266
	KNN	0.7328 ± 0.0389	0.7246 ± 0.0369	0.7226 ± 0.0373	0.7252 ± 0.0368
	NB	0.6869 ± 0.0454	0.6810 ± 0.0440	0.6789 ± 0.0447	0.6815 ± 0.0440
	DT	0.6275 ± 0.0441	0.6270 ± 0.0443	0.6264 ± 0.0449	0.6270 ± 0.0443
	MLP	0.8052 ± 0.0201	0.8036 ± 0.0213	0.8033 ± 0.0215	0.8036 ± 0.0211
EfficientNetB0	SVM	**0.8362 ± 0.0363**	**0.8335 ± 0.0377**	**0.8330 ± 0.0383**	**0.8334 ± 0.0379**
	RF	0.7630 ± 0.0569	0.7601 ± 0.0576	0.7592 ± 0.0583	0.7600 ± 0.0578
	XGBoost	0.7906 ± 0.0589	0.7886 ± 0.0594	0.7882 ± 0.0596	0.7887 ± 0.0595
	LR	0.8117 ± 0.0419	0.8086 ± 0.0421	0.8080 ± 0.0424	0.8086 ± 0.0421
	KNN	0.7635 ± 0.0382	0.7595 ± 0.0381	0.7589 ± 0.0385	0.7600 ± 0.0380
	NB	0.6688 ± 0.0658	0.6656 ± 0.0657	0.6640 ± 0.0660	0.6657 ± 0.0657
	DT	0.6331 ± 0.0389	0.6309 ± 0.0391	0.6292 ± 0.0398	0.6310 ± 0.0392
	MLP	**0.8353 ± 0.0483**	**0.8335 ± 0.0479**	**0.8332 ± 0.0479**	**0.8334 ± 0.0479**

**Table 4:** Performance evaluation metrics obtained from vision transformer (ViT) models.

Model	Classifier	Performance Evaluation Metrics			
		Precision	Recall	F1 Score	Accuracy
ViT Base Patch16-224	SVM	0.8041 ± 0.0387	0.8018 ± 0.0386	0.8012 ± 0.0387	0.8016 ± 0.0385
	RF	0.7454 ± 0.0320	0.7442 ± 0.0317	0.7437 ± 0.0318	0.7440 ± 0.0318
	XGBoost	0.7701 ± 0.0333	0.7658 ± 0.0325	0.7649 ± 0.0325	0.7659 ± 0.0324
	LR	0.7969 ± 0.0347	0.7956 ± 0.0349	0.7954 ± 0.0351	0.7956 ± 0.0350
	KNN	0.7375 ± 0.0325	0.7338 ± 0.0315	0.7331 ± 0.0314	0.7342 ± 0.0314
	NB	0.7081 ± 0.0478	0.7057 ± 0.0468	0.7046 ± 0.0467	0.7053 ± 0.0468
	DT	0.6362 ± 0.0546	0.6359 ± 0.0546	0.6358 ± 0.0546	0.6361 ± 0.0546
	MLP	0.8098 ± 0.0322	0.8086 ± 0.0320	0.8084 ± 0.0320	0.8085 ± 0.0320
ViT Base Patch32-224	SVM	0.8148 ± 0.0460	0.8117 ± 0.0465	0.8110 ± 0.0466	0.8115 ± 0.0464
	RF	0.7663 ± 0.0413	0.7649 ± 0.0411	0.7646 ± 0.0410	0.7649 ± 0.0409
	XGBoost	0.7709 ± 0.0538	0.7669 ± 0.0561	0.7657 ± 0.0570	0.7669 ± 0.0561
	LR	0.7864 ± 0.0411	0.7817 ± 0.0427	0.7807 ± 0.0434	0.7817 ± 0.0428
	KNN	0.7759 ± 0.0437	0.7716 ± 0.0446	0.7707 ± 0.0450	0.7718 ± 0.0445
	NB	0.7323 ± 0.0429	0.7284 ± 0.0435	0.7269 ± 0.0441	0.7282 ± 0.0436
	DT	0.6392 ± 0.0305	0.6367 ± 0.0302	0.6351 ± 0.0305	0.6369 ± 0.0299
	MLP	0.8290 ± 0.0355	0.8244 ± 0.0387	0.8236 ± 0.0395	0.8244 ± 0.0387
ViT Base Patch 16-384	SVM	0.8176 ± 0.0420	0.8147 ± 0.0434	0.8110 ± 0.0466	0.8145 ± 0.0433
	RF	0.7820 ± 0.0293	0.7808 ± 0.0295	0.7805 ± 0.0297	0.7808 ± 0.0296
	XGBoost	0.7963 ± 0.0400	0.7928 ± 0.0408	0.7920 ± 0.0410	0.7927 ± 0.0407
	LR	0.8099 ± 0.0420	0.8067 ± 0.0438	0.8059 ± 0.0446	0.8065 ± 0.0440
	KNN	0.7807 ± 0.0241	0.7789 ± 0.0251	0.7784 ± 0.0255	0.7788 ± 0.0252
	NB	0.7242 ± 0.0399	0.7204 ± 0.0370	0.7193 ± 0.0366	0.7202 ± 0.0370
	DT	0.6509 ± 0.0684	0.6500 ± 0.0678	0.6495 ± 0.0677	0.6499 ± 0.0677
	MLP	**0.8494 ± 0.0309**	**0.8464 ± 0.0328**	**0.8458 ± 0.0334**	**0.8462 ± 0.0330**
ViT Base Patch32-384	SVM	0.8264 ± 0.0365	0.8245 ± 0.0373	0.8240 ± 0.0374	0.8243 ± 0.0372
	RF	0.7779 ± 0.0422	0.7759 ± 0.0418	0.7754 ± 0.0417	0.7758 ± 0.0416
	XGBoost	0.7823 ± 0.0331	0.7799 ± 0.0348	0.7792 ± 0.0353	0.7798 ± 0.0348
	LR	0.8264 ± 0.0347	0.8223 ± 0.0365	0.8217 ± 0.0370	0.8223 ± 0.0364
	KNN	0.7881 ± 0.0231	0.7816 ± 0.0244	0.7803 ± 0.0251	0.7817 ± 0.0244
	NB	0.7388 ± 0.0270	0.7373 ± 0.0269	0.7367 ± 0.0269	0.7371 ± 0.0268
	DT	0.6628 ± 0.0504	0.6617 ± 0.0501	0.6611 ± 0.0502	0.6618 ± 0.0502
	MLP	0.8459 ± 0.0276	0.8403 ± 0.0290	0.8395 ± 0.0293	0.8402 ± 0.0289

## Data Availability

The datasets used and/or analyzed during the current study are available from the corresponding author on reasonable request.

## Abbreviations

AI	Artificial intelligence
AUC	Area under the curve
CBCT	Cone beam computed tomography
CNN	Convolutional neural network
KNN	K-nearest neighbors
LR	Logistic regression
OPG	Panoramic radiography
RF	Random forest
ROI	Region of interest
SVM	Support vector machine
ViT	Vision transformer
TP	True positive
TN	True negative
FP	False positive
FN	False negative
PR	Precision–recall

## References

[1] Yu X., Guo B., Li K.Z., Zhang R., Tian Y.Y., Wang H., Dds T.H. (2012). Cone-beam computed tomography study of root and canal morphology of mandibular premolars in a western Chinese population. BMC Med. Imaging.

[2] Shabahang S. (2013). Treatment Options: Apexogenesis and Apexification. J. Endod..

[3] Proffit W.R., Fields H., Larson B., Sarver D.M. (2019). Contemporary Orthodontics, 6e: South Asia Edition-E-Book.

[4] Lin Y., Thumbigere-Math V., Kishen A., He J. (2025). Unraveling the Etiology and Pathogenesis of Multiple Cervical Root Resorption—A Scoping Review. J. Endod..

[5] Caeiro-Villasenín L., Serna-Muñoz C., Pérez-Silva A., Vicente-Hernández A., Poza-Pascual A., Ortiz-Ruiz A.J. (2022). Developmental Dental Defects in Permanent Teeth Resulting from Trauma in Primary Dentition: A Systematic Review. Int. J. Environ. Res. Public Health.

[6] Wilson Y.P., Nambiar P., Yaacob H., Asif M.K. (2021). Age estimation from developing third molars. Med. Leg. J..

[7] Patel P., Shah J., Dudhia B., Butala P., Jani Y., Macwan R. (2020). Comparison of panoramic radiograph and cone beam computed tomography findings for impacted mandibular third molar root and inferior alveolar nerve canal relation. Indian J. Dent. Res..

[8] Kalabalık F., Yılmaz N., Aydın E.G., Aytuğar E. (2024). Investigation of root apical closure of first permanent molars with cone-beam computed tomography: A retrospective study. J. Dent. Sci..

[9] Stera G., Giusti M., Magnini A., Calistri L., Izzetti R., Nardi C. (2024). Diagnostic accuracy of periapical radiography and panoramic radiography in the detection of apical periodontitis: A systematic review and meta-analysis. Radiol. Med..

[10] Ghasemi N., Rokhshad R., Zare Q., Shobeiri P., Schwendicke F. (2025). Artificial intelligence for osteoporosis detection on panoramic radiography: A systematic review and meta analysis. J. Dent..

[11] Ketenci Çay F., Yeşil Ç., Çay O., Yılmaz B.G., Özçini F.H., İlgüy D. (2025). DeepLabv3+ method for detecting and segmenting apical lesions on panoramic radiography. Clin. Oral Investig..

[12] Schwendicke F., Elhennawy K., Paris S., Friebertshäuser P., Krois J. (2020). Deep learning for caries lesion detection in near-infrared light transillumination images: A pilot study. J. Dent..

[13] Talu M.H., Baybars Coşgun S., Danacı Ç., Aslan Tuncer S. (2025). From Image to Diagnosis: Convolutional Neural Networks in Tongue Lesions. J. Imaging Inform. Med..

[14] Bansal R.K., Arya A., Singh B., Singla M., Gupta S. (2025). Role of Artificial Intelligence and Machine Learning in Conservative Dentistry and Endodontics: A Review. Cureus.

[15] Dosovitskiy A., Beyer L., Kolesnikov A., Weissenborn D., Zhai X., Unterthiner T., Dehghani M., Minderer M., Heigold G., Gelly S. (2021). An image is worth 16X16 words: Transformers for image recognition at scale. arXiv.

[16] Khan S., Naseer M., Hayat M., Zamir S.W., Khan F.S., Shah M. (2022). Transformers in Vision: A Survey. ACM Comput. Surv..

[17] Han K., Wang Y., Chen H., Chen X., Guo J., Liu Z., Tang Y., Xiao A., Xu C., Xu Y. (2023). A Survey on Visual Transformer. IEEE Trans. Pattern Anal. Mach. Intell..

[18] Zhang X., Guo E., Liu X., Zhao H., Yang J., Li W., Wu W., Sun W. (2025). Enhancing furcation involvement classification on panoramic radiographs with vision transformers. BMC Oral Health.

[19] Kahm S.H., Kim J.Y., Yoo S., Bae S.M., Kang J.E., Lee S.H. (2023). Application of entire dental panorama image data in artificial intelligence model for age estimation. BMC Oral Health.

[20] Hayat M., Aramvith S., Bhattacharjee S., Ahmad N. (2025). Attention GhostUNet++: Enhanced Segmentation of Adipose Tissue and Liver in CT Images. arXiv.

[21] Hayat M., Gupta M., Suanpang P., Nanthaamornphong A. (2024). Super-Resolution Methods for Endoscopic Imaging: A Review. Proceedings of the 2024 12th IEMECON.

[22] Shobhana R., Prakash V., Venkatesh A., Vivekanandhan P. (2021). A Review on Management of Open Apex in Permanent Teeth. Int. J. Aquat. Sci..

[23] Andreasen J.O., Bakland L.K., Matras R.C., Andreasen F.M. (2006). Traumatic intrusion of permanent teeth. Part 1. An epidemiological study of 216 intruded permanent teeth. Dent. Traumatol..

[24] Kumar S., Kumar T., Keshav V., Arora S., Singla A. (2019). Open apex solutions: One-step apexification, salvaging necrosed teeth with open apex. Endodontology.

[25] Saghiri M.A., Asgar K., Boukani K.K., Lotfi M., Aghili H., Delvarani A., Karamifar K., Saghiri A.M., Mehrvarzfar P., Garcia-Godoy F. (2012). A new approach for locating the minor apical foramen using an artificial neural network. Int. Endodontic. J..

[26] Wang X., Alqahtani K.A., Van Den Bogaert T., Shujaat S., Jacobs R., Shaheen E. (2024). Convolutional neural network for automated tooth segmentation on intraoral scans. BMC Oral Health.

[27] Kora P., Ooi C.P., Faust O., Raghavendra U., Gudigar A., Chan W.Y., Meenakshi K., Swaraja K., Plawiak P., Acharya U.R. (2022). Transfer learning techniques for medical image analysis: A review. Biocybern. Biomed. Eng..

[28] Mun S.B., Kim J., Kim Y.J., Seo M.S., Kim B.C., Kim K.G. (2024). Deep learning-based prediction of indication for cracked tooth extraction using panoramic radiography. BMC Oral Health.

[29] Pirayesh Z., Mohammad-Rahimi H., Motamedian S.R., Amini Afshar S., Abbasi R., Rohban M.H., Mahdian M., Ahsaie M.G., Alamdari M.I. (2024). A hierarchical deep learning approach for diagnosing impacted canine-induced root resorption via cone-beam computed tomography. BMC Oral Health.

[30] Yuan L., Chen Y., Wang T., Yu W., Shi Y., Jiang Z., Tay F.E.H., Feng J., Yan S. (2021). Tokens-to-Token ViT: Training Vision Transformers from Scratch on ImageNet. arXiv.

[31] Shisu Y., Mingwin S., Wanwag Y., Chenso Z., Huing S. (2024). Improved EATFormer: A Vision Transformer for Medical Image Classification. arXiv.

[32] Ahmed M.d.M., Hossain M.d.M., Islam M.d.R., Ali M.d.S., Nafi A.A.N., Ahmed M.d.F., Ahmed K.M., Miah S., Rahman M., Niu M. (2024). Brain tumor detection and classification in MRI using hybrid ViT and GRU model with explainable AI in Southern Bangladesh. Sci. Rep..

[33] Yang X., Li X., Li X., Wu P., Shen L., Deng Y. (2024). ImplantFormer: Vision Transformer based Implant Position Regression Using Dental CBCT Data. Neural Comput. Appl..

[34] Felsch M., Meyer O., Schlickenrieder A., Engels P., Schönewolf J., Zöllner F., Heinrich-Weltzien R., Hesenius M., Hickel R., Gruhn V. (2023). Detection and localization of caries and hypomineralization on dental photographs with a vision transformer model. npj Digit. Med..

[35] Dujic H., Meyer O., Hoss P., Wölfle U.C., Wülk A., Meusburger T., Meier L., Gruhn V., Hesenius M., Hickel R. (2023). Automatized Detection of Periodontal Bone Loss on Periapical Radiographs by Vision Transformer Networks. Diagnostics.

